# Adipocyte-Based Cell Therapy in Oncology: The Role of Cancer-Associated Adipocytes and Their Reinterpretation as Delivery Platforms

**DOI:** 10.3390/pharmaceutics12050402

**Published:** 2020-04-28

**Authors:** Raluca Munteanu, Anca Onaciu, Cristian Moldovan, Alina-Andreea Zimta, Diana Gulei, Angelo V. Paradiso, Vladimir Lazar, Ioana Berindan-Neagoe

**Affiliations:** 1 Research Center for Advanced Medicine-Medfuture, Iuliu Hatieganu University of Medicine and Pharmacy, 23 Marinescu Street, 400337 Cluj-Napoca, Romania; 2 Oncologia Sperimentale, Istituto Tumori G Paolo II, IRCCS, 70125 Bari, Italy; 3 Worldwide Innovative Network for Personalized Cancer Therapy, 94800 Villejuif, France; 4 Research Center for Functional Genomics, Biomedicine and Translational Medicine, Iuliu Hatieganu University of Medicine and Pharmacy, 23 Marinescu Street, 400337 Cluj-Napoca, Romania; 5 Department of Functional Genomics and Experimental Pathology, The Oncology Institute “Prof. Dr. Ion Chiricuta”, 34-36 Republicii Street, 400015 Cluj-Napoca, Romania

**Keywords:** adipocytes, delivery, cancer therapy, exosomes

## Abstract

Cancer-associated adipocytes have functional roles in tumor development through secreted adipocyte-derived factors and exosomes and also through metabolic symbiosis, where the malignant cells take up the lactate, fatty acids and glutamine produced by the neighboring adipocytes. Recent research has demonstrated the value of adipocytes as cell-based delivery platforms for drugs (or prodrugs), nucleic acids or loaded nanoparticles for cancer therapy. This strategy takes advantage of the biocompatibility of the delivery system, its ability to locate the tumor site and also the predisposition of cancer cells to come in functional contact with the adipocytes from the tumor microenvironment for metabolic sustenance. Also, their exosomal content can be used in the context of cancer stem cell reprogramming or as a delivery vehicle for different cargos, like non-coding nucleic acids. Moreover, the process of adipocytes isolation, processing and charging is quite straightforward, with minimal economical expenses. The present review comprehensively presents the role of adipocytes in cancer (in the context of obese and non-obese individuals), the main methods for isolation and characterization and also the current therapeutic applications of these cells as delivery platforms in the oncology sector.

## 1. Introduction

Malignant pathologies are in the frontline of research due to the high incidence and mortality rates observed worldwide [1]. The main subject approaches are related to early and minimally invasive diagnosis in order to detect the disease in incipient stages and obtain a good treatment evolution and new and improved therapeutic approaches for late-stage and therapy-resistant malignancies. The last subject involves, besides the formulation of new therapeutics, advanced delivery platforms able to concentrate the drug at the tumor site and also cause minimal side effects [2,3,4]. In these terms, cell delivery systems are of interest due to high biocompatibility, long circulation when injected systemically, tropism toward tumors and inflammatory sites [5].

Adipocytes are naturally present in the human organism and their isolation and characterization is straightforward, involving minimal invasive procedures and also quite economical techniques. Moreover, their lipid content is an ideal media for encapsulation of hydrophobic drugs. The accumulation of lipid droplets is engaged in the lipolysis process triggered by tumor cells in order to sustain their metabolism, a process that can be used as a Trojan horse strategy for local and sustained delivery of different drugs at the tumor site [6].

Current data shows that adipose-derived stem cells (ADSCs) can stimulate tumor growth in different pathologies such as lung [7], breast [8], thyroid [9] or colon cancer [10]. On the other hand, the evidence shows that ADSCs also have the ability to inhibit tumor development. In vivo and in vitro studies should only be performed with highly encouraging results, and in this regard, some studies have highlighted the cancer inhibition potential of adipose stem cells. For example, two different prostate cancer cell lines were analyzed, one androgen-responsive and one non-responsive, and local transplantation of ADSCs proved to considerably reduce the tumor growth progression rate [11]. Also, some of these adipose systems were developed using their capacity as carriers for different structures, such as nanoparticles (NPs) or viruses. A recent study carried out by Huang et al. [12] focuses on brain tumors, and the vehicle property of adipocytes stem cells is engaged to deliver different NPs or compounds, such as nucleic acids, to the tumoral site.

Despite the potential advantages, the use of these cell-based delivery systems is disputed, and there is a need for more sophisticated clinical studies within in vivo subjects to confirm utility.

The benefits of human ADSC (hADSC) platforms have also been studied in other fields, especially in regenerative medicine. Renae et al. [13] based their research on an injectable hydrogel together with hADSC secretome. The purpose was to obtain peri-infarct myocardium delivery based on a nanocomposite hydrogel system. This method improved cardiac function, observed in vivo by comparing the secretome-loaded hydrogel group with the control group, and finally also led to the diminution of scarring tissue. These types of strategies can in part be recapitulated in the oncology field in terms of cell processing and manipulation.

This review presents the field of adipocytes-based delivery in cancer by summarizing firstly their role in cancer installation and development (obese and non-obese individuals), the methods of isolation and characterization, conjugation strategies involving genetic engineering, drug loading or NP fusion, and also current applications for cancer treatment.

## 2. Current Cell-Based Approaches in Drug Delivery

Existing pharmaceutical compounds have the capacity to induce important effects in various diseases. However, in order to achieve their purpose in patients with maximal efficacy, they need to accumulate at the target site at high concentrations and overcome biological barriers that include degradation and immune clearance [14,15].

Targeted therapy aims to bypass these obstacles by relying on vehicles able to carry active compounds to specific cells/tissues. The most common carriers used for targeted drug delivery are synthetic NPs, which seem to have gained ground lately due to their interesting physico-chemical properties and possibility to easily design them to have specificity for unique sites.

It has been described that systemically administered NPs accumulate passively in tumors or inflamed sites of mouse models, due to the enhanced permeability and retention effect (EPR). Nonetheless, on average, less than 1% of the injected NPs can be detected in tumor sites [16], which is inefficient since the rest could be distributed throughout healthy tissues and cells, inducing negative effects. Active targeting relies on the functionalization of NPs with ligands such as small molecules, peptides, aptamers or antibodies, which can specifically bind epitopes that are overexpressed or exclusively expressed by the target sites [16]. Moreover, other approaches to ensure the bioavailability of the drugs at target sites rely on NPs that are sensitive to external stimuli, such as superparamagnetic NPs [17] or nanocarriers with stimuli-dependent content release, where, for example, the acidic pH of the tumor microenvironment induces the drug release [18]. However, the complicated carrier design and synthesis are big drawbacks for these strategies.

Due to the numerous limitations of synthetic NPs, including the ones mentioned above, cell-based and cell-derivates-based drug carriers have gained significant attention lately [19] for use in various diseases, including cancer, diabetes and autoimmune diseases.

### Cell Types Used as Drug Delivery Vehicles

Various cell types present unique and attractive features which make them suitable for use as drug delivery systems. Depending on the cell type, their advantages vary in terms of mobility, circulation lifespan duration, biodegradability and biocompatibility, interaction with other cells and molecules, drug loading capacity and the ability to overcome biological barriers [20]. This makes specific cells superior to others for use as drug carriers depending on the pathology taken into account.

The most commonly used cells as drug delivery systems, the aspects that make them suitable for this purpose, the pathology they are used in and the loaded drugs are presented in Table 1.

The majority of clinical trials involving the use of adipocytes are focused on regenerative medicine; however, these cells have also been adapted for other studies, including cancer treatment. Autologous fat tissue research has been conducted to the development of clinical trials with representative results in plastic and reconstruction surgery and regenerative medicine, especially for: breast augmentation [55,56], breast implants preventing complications [57], breast reconstruction deformities correction [56,58], soft tissue augmentation [59,60], supporting fat tissue transplantation [61], facial rejuvenation [62], burns regeneration [63] and fistulas reparation [64]. In addition, some of the clinical trials were orientated in using adipose tissue cells as treatment of various pathologic conditions: degenerative disk disease [65], ischemic disease [66], scarred vocal folds [67] and male stress urinary incontinence [68].

## 3. The Role of Adipocytes in Cancer

### 3.1. The Environment of the Adipose Tissue

Initially considered a cell with roles just in energy storage, the adipocyte is now known as a complex cellular entity with additional roles involving endocrine and paracrine signaling through secreted adipokines. In this way, the adipose tissue is functionally interconnected with peripheral organs and systems, such as the liver and hypothalamus, but also sustains a local interaction between neighboring adipocytes and other types of local cells [69]. Specific functions attributed to the adipose tissue consist in the expenditure of the body energy and also food intake behavior, immune functions, reproduction and hematopoiesis and lymphopoiesis. Moreover, it is actively involved in the installation and development of pathological states, including cancer (subject discussed in the next subchapter). The activity of the adipose tissue depends also on the location within the organisms (visceral, subcutaneous, intramuscular) and specific subtype (white, brown, or beige adipose tissue). In non-obese humans, the majority of the adipose tissue consists in subcutaneous fat that represents approximately 80% of the depots, followed by visceral adipocytes (5–20%) and multiple small depots found at the bone marrow and at the intramuscular and intraorbital sites [70]. In terms of tissue subtypes, the majority of the depots are composed of white adipose tissue, completed by small islands of brown and beige ones in adults [71,72].

Although in standard histological sections the adipose tissue appears quite homogenous in terms of cellular composition (mainly adipocytes—90% of the tissue volume), it also comprises additional cells that form the stromal-vascular fraction represented by endothelial cells, fibroblasts together with other connective tissue cells, pericytes, progenitor and stem cells, and cells of the immune system (macrophages, mast cells, dendritic cells, neutrophils, eosinophils and lymphocytes) with functional roles within the tissue [73]. For example, in macrophages’ infiltration, it is accentuated around necrotic adipocytes, found especially in obese organisms, with roles in clearance of the residual “free” adipocyte lipid droplet. The “excessive” clearance determines the formation of macrophage syncytia and multinucleate giant cells that are causing chronic inflammation and implicit metabolic complications. This “excessive” clearance is correlated with an increase in the size of the adipose tissue and implicitly with a high number of apoptotic adipocytes, all parameters of an obese individual [74]. Endothelial cells found within the adipocyte mass have a functional role in vasculature formation with consequences upon development and adipose tissue expansion [75]. Subsequently, it was shown that adipose cells and endothelial cells are able to crosstalk through the release of extracellular vesicles. Moreover, the endothelial cells are able to secrete PPARγ (peroxisome proliferator-activated receptor-γ) ligands, where PPARγ is one of the essential regulators of lipids’ storing in adipocytes and also their differentiation. While adipocytes do not have the ability to secrete endogenous PPARγ ligands, they depend on the crosstalk with the endothelial cells within the adipose tissue to coordinate in the regulation of lipid uptake [76]. Adipose tissue stem cells (ASCs) from stromal vascular fractions (SVF) of adipose tissue have the capacity to differentiate in multiple cell lineages (e.g., adipocytes, chondroblasts, osteoblasts, myocytes and cardiomyocytes—data shown in vitro [77]) and have represented an attractive therapeutic option since early 2007, especially in the sector of regenerative medicine. In line with the topic of the present paper, referring to the capacity of adipocytes to function as delivery platforms for anticancer drugs, the subject of ASCs used as therapeutic agents for regenerative medicine and also other health applications could be of interest. The technical challenges, routes of administration and also the behavior of ASCs or more differentiated cells originating from the adipose tissue encountered in current experimental approaches could sustain their application as drug delivery platforms by learning from overlapping technical steps. ASCs can be relatively easily isolated from the adipose tissue of the patient and transplanted back at the target site through direct injection or administrated through the bloodstream (in free form or encapsulated in different biomaterials). Although the therapeutic role of these kind of strategies consists in an increase of the healing rate through liberation of cytokines and growth factors with effects on the affected environment [78], ASC can also be used in a Trojan horse strategy to deliver cytotoxic agents to tumor cells.

The most important characteristics of the human adipose tissue are summarized in Table 2.

### 3.2. The Link between Adipose Tissue and Cancer

Cancer represents one of the leading pathologies in terms of incidence and mortality rates at the global level, being situated in the top after heart diseases. Among the main contributors to the high mortality rates are the late diagnosis, development of metastasis and installation of drug resistance. Even so, an important part of cancer management is represented by the delivery of the cytotoxic agents that at the tumor site should ideally bring an increased concentration of drug, in a targeted manner, without affecting the normal cells or without losing concentration in the systemic pathway. In this context, numerous studies are focusing on different delivery vehicles represented by synthetic materials or biological ones, where adipocytes represent a novel approach for such strategies, taking advantage of the relatively easy harvesting and processing steps and also the biological compatibility and functional role within the tumor that “tricks” the malignant cells.

The majority of studies focusing on the adipose tissue and cancer highlight the link between obesity and installation of malignant pathologies, together with the prognosis of such patients. Most of the studies are supporting their hypotheses on observational facts based on large cohorts that are sometimes difficult to interpret due to heterogeneity in patients’ clinical and demographic parameters; however, the link between obesity and cancer is supported by consistent evidence [15,79], especially in the case of: endometrial cancer [80,81], esophageal adenocarcinoma [82], gastric cardia cancer [83], liver cancer [84,85], kidney cancer [86,87], multiple myeloma [88], meningioma [89], pancreatic cancer [90], gallbladder cancer [91], breast cancer [92,93,94], ovarian cancer [95] and thyroid cancer [96]. The National Cancer Institute (NCI) proposes several mechanisms behind the association that consists in chronic local inflammation in obese individuals, that, during time, can determine modifications at the DNA level, increased amount of estrogen produced by the adipose tissue that can determine the installation of hormone-dependent malignancies, high level of insulin and insulin-like growth factor-1 (IGF-1) in the blood associated with installation of prostate, endometrial, kidney and colon cancer, increased secretion of adipokines with roles in cell proliferation (e.g., leptin) and the indirect effect of adipocytes in modulating other signaling pathways involved in cell growth (e.g., mammalian target of rapamycin—mTOR and AMP-activated protein kinase—AMPK) [97]. The evolution of obese cancer patients has been monitored during observational studies that show a worse prognosis and survival rate and also increased changes of recurrence and progression compared to those with normal weight [98,99]. There is a continuous effort in understanding the molecular aspects behind this pathological association, including the role of gut microbiota and the role of insulin receptor signaling; however, the purpose of our study is to present the role of the adipose tissue per se in the co-evolution with cancer in order to highlight both types of cell behavior and to identify the interactions between adipocytes and tumor cells that can be exploited in experimental adipocyte-based drug delivery strategies. It is important to mention that the study of adipocytes in the context of non-obese cancer patients or animal models is far less extensive then the ones associated with obesity, a fact that hinders the translational value for adipocyte-based drug delivery. This method of delivery could have a significant impact in the clinic but is dependent on basic science studies focused on the role of adipocytes in different malignancies (it is important that cancer cells establish a functional connection with the engineered adipocyte in order to come in contact with the encapsulated drug). After studying the current literature, it was observed that breast and prostate cancer are the two malignancies with the most data in the field (these tumor sites are also suitable for intratumor injection of adipocytes). Table 3 outlines the most important characteristics of the connection between adipose tissue and cancer that are presented in detail within the text.

**Figure 1:**
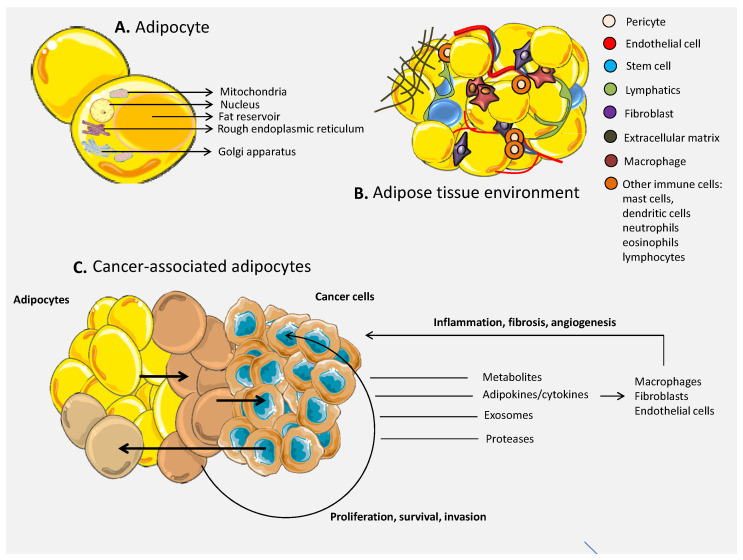
The composition and role of adipose tissue in cancer development. (**A**) Adipocytes are complex cellular entities containing nucleus, mitochondria, Golgi apparatus and rough endoplasmic reticulum and the fat reservoir that occupies the majority of the cellular space. (**B**) The adipose tissue is composed of, besides adipocytes, numerous cell types, including: endothelial cells, pericytes, stem cells fibroblasts and cells of the immune system, all of which have functional roles in the tissue homeostasis. (**C**) Adipocytes, upon malignant pressure, can switch into cancer-associated adipocytes (CAAs) that contribute to the installation and development of the tumor mass through secreted metabolites, adipokines/cytokines, exosomes and proteases, with roles in cell proliferation, survival and invasion. The influence of the secreted adipokines/cytokines is also retained on the cells of the tumor environment cells, sustaining processes like inflammation, angiogenesis and fibrosis.

Cancer is a microenvironment-dependent pathology. Part of it is represented by cancer-associated adipocytes (CAAs) that can be found neighboring the malignant cells, forming the tumor margins or infiltrating in the malignant body. Adipocytes are associated with a functional role in tumor progression through paracrine and endocrine influence on tumor cells through secreted adipocyte-derived factors [73] (Figure 1). Adiponectin and leptin are true adipokines, while other signaling molecules are attributed to both adipocytes and immune cells: tumor necrosis factor alpha (TNF-α), interleukin-6 (IL-6), resistin, visfatin, chemokine monocyte chemoattractant protein (MCP-1) and plasminogen activator inhibitor-1 (PAI-1) [100]. Leptin is expressed from the obese (*ob*) gene and is responsible for the distribution of energy, control of body weight and regulation of appetite. While in mice, mutations of the *ob* gene determines obesity, infertility, diabetes and hypothermia [101], in humans, the installation of obesity is not related to a lack of functional leptin, but rather leptin resistance (*ob* mutations are very rare in humans) [102,103,104,105]. Secretion of leptin by adipocytes is in turn modulated by TNF-α, insulin, glucocorticoids, prostaglandins and reproductive hormones [106]. In cancer, the installation of hypoxic conditions is a driver of leptin secretion through hypoxia-induced factor-1 (HIF-1) that activates the leptin gene promoter in adipocytes and fibroblasts [107,108]. Moreover, leptin is involved in vascular permeability and remodeling alone or in combination with vascular endothelial growth factor (VEGF) and fibroblast growth factor (FGF) 2 [109]. Leptin inhibits cell death and stimulates endothelial cell growth in a Bcl-2-dependent strategy [110], contributing to the proangiogenic activity that can be recapitulated in cancer. Studies showed that the same adipokine sustains the proliferation of breast cancer cells [111] and positively influences the growth of neoplastic colon cells [112]. Cancer cells trap the influence of leptin through overexpression of the leptin receptor (Ob-R) that is normally expressed predominantly in the hypothalamus and at lower levels in other parts of the body (e.g., breast epithelial cells and pancreas) [113]. Research has shown that leptin is also involved in chemoresistance through induction of ATP-binding cassette (ABC) protein transporters (in glioblastoma, breast and pancreatic cancer) [113,114] and activation of NFκB signaling under treatment [115]. Complex feedback mechanisms are found in breast cancer, where leptin activates Oct-4 and Nanog, which in turn increase the expression of Ob-R in malignant cells [113]. Altogether, the adipokine is becoming an attractive therapeutic target in cancer. In the context of the present study, the adipocytes for drug delivery could also be engineered to not express leptin in order to decrease their positive impact upon the tumor mass and to increase the efficiency of the encapsulated therapeutic agent. However, the effect of resident CAAs will still be present and their impact probably depends on the number of engineered adipocytes injected at the tumor sites and their capacity to take over the communication with the tumor. In contrast, adiponectin (APN) has been mainly associated with anti-carcinogenic effects via modulation of apoptosis, cell survival and metastasis, although there are some studies sustaining a contradictory effect [116]. The main concentration of APN comes from the white adipose tissue and also in smaller quantities from the brown one. In general, decreased levels of APN have been observed in several cancers, but it is important to take into consideration the distinct isoforms that could sustain different functions (more details reviewed by Katira et al. [116]). Acute lymphoblastic leukemia (ALL) pediatric patients exhibit high levels of leptin and low levels of APN at the diagnosis, while the balance of the adipokines progressively return to homeostatic values during therapy, representing a sign of good health [117]. APN is also associated with the ability to suppress the metastasis of breast cancer through a liver kinase B1 (LKB1)-mediated signaling [118,119] and the AMPK/Akt pathway [120]. APN is able to impair the invasion sustained by leptin through inactivation of the JAK/STAT3 pathway and stimulation of AMPK signaling in endometrial cancer cells. Cancer-specific APN research has been reviewed in more detail by Katira et al. [116]. In the light of engineered adipocytes, artificial increase of APN secretion could sustain the effect of the encapsulated drug (an effect that could be more pronounced by a concomitant decrease of leptin in the same cells). However, for this possible strategy, it is important to consider the contribution to the serum levels of other adipocytes not present at the tumor level (an effect that should be less pronounced in non-obese individuals and more restrictive to CAA).

Malignant cells are also dependent on the CAA due to the metabolic symbiosis that is forming between the two types of cells during cancer progression. Specifically, hypoxic tumor cells turn to lactate, fatty acids and glutamine for their energy sources that are liberated into the environment by adipocytes [121,122,123] or other cancer cells [124,125]. This feature is essential in the Trojan horse strategy with engineered adipocytes in delivering cytotoxic drugs to the tumor. As an example, successive clinical evidence shows a metastasis preference of ovarian cancer to the omentum, an organ formed mainly by adipocytes, and co-culture of ovarian cancer cells with adipocytes highlights the direct lipid transfer toward the malignant ones together with an increase in tumor growth both in vitro and in vivo. Higher levels of fatty acid-binding protein 4 (FABP4) are found in the omental metastases in comparison with the cells from the primary tumors, a protein that was also found at the adipocyte–tumor cell interface. Experimental decrease of FABP4 impaired the metastatic dissemination of ovarian cancer cells in vivo [121]. Also, adipocytes rescued pancreatic cancer cells in in vitro media with minimal nutrients (0.5% FCS (Fetal Calf Serum), 0 mM glucose, 0 mM glutamine) through glutamine transfer activity, dependent on glutamine synthetase and glutaminase modified balance in adipocytes. No significant impact was seen in glucose shuttling for sustenance of cancer cells proliferation [123]. Importantly for adipocyte-mediated drug delivery, the effect of pancreatic cancer cells’ rescue was greater in the case of adipocytes than preadipocytes, where the latter have significantly less lipid stores, the most probable source of glutamine.

The communication between CAA and malignant cells is sustained by exosomes, as shown by Lazar et al. [126] in the case of invasive melanoma. These nanovesicles are able to transfer functional material over short or long distances, with further molecular and behavioral effects on the receiving cells. Malignant cells become more invasive and have enhanced migration abilities after taking up the exosomes secreted by adipocytes. The proteome of these exosomes show a predominant signature of proteins with roles in fatty acid oxidation (FAO), and a metabolic pathway that is increased in melanoma cells once in contact with these exosomes [126]. MSC-differentiated adipocyte-derived exosomes sustain the migration and proliferation of breast cancer cells after active incorporation, and also exert chemotherapeutic protective effects. Transcriptomics profiling identified the Hippo signaling pathway as one of the main pathways responsible for the cancer promoting effects, where its abrogation was associated with reduced exosomes-induced tumor growth [78].

### 3.3. Cells of the Immune System within the Adipose Tissue in Cancer

Adipocytes establish many interactions with the immune cells at the tumor site that are important for the sustenance of tumor growth through local chronic inflammation. They secrete chemokines, such as CCR2 and CCR4, that bind to CCL2. The activation of CCL2 in circulating monocytes attracts them at the tumor site, and in breast cancer cells, it induces the formation of cancer stem cells (CSCs) through activation of the Notch signaling pathway [127]. Cenicriviroc is an inhibitor of CCR2 that has high affinity for the adipose tissue and that has been tested as a drug option against nonalcoholic steatohepatitis in mice. This drug is also able to prevent monocyte recruitment in the case of this disease [128]. Theoretically, an investigation of cenicriviroc loading of CAAs and delivery in breast, ovarian or prostate cancer would be worth investigating.

Another aspect of adipocytes’ modulation of tumor immunity is their ability to express PDL1 on their surface, adding to the PDL1 immunosuppressive effects from cancer cells, thus leading to exacerbated T cell exhaustion. The expression of PDL1 shows a progressive increase as the pre-adipocytes differentiate into adipocytes. Also, the expression of PDL1 is more overexpressed in brown adipose tissue than in white adipose tissue. This is especially important for the newly developed PD1-PDL1 therapy and why it fails in some types of cancer, such as breast cancer. In order to put this theory to the test, a group of researchers tested anti-PDL1 therapy on splenocytes. They concluded that the therapy is very effective in simple splenocytes, while in adipocytes co-cultured with splenocytes, it has no effect. The adipocytes’ activities in anti-PDL1 therapy most probably has three ways of action: (1) competitive inhibition for PDL1 between adipocytes and cancer cells, (2) direct contact with T-cells that by-passed cancer cell interaction with T cells and (3) PDL1 is localized intracytoplasmatically in a small population of adipocytes, thus being protected from anti-PDL1 antibody-based therapy [129].

As stated earlier, adipocytes secrete true adipokines (adiponectin and leptin), but also signaling molecules attributed to both adipocytes and immune cells [100]. The co-culture of CAAs with breast cancer cells increases the secretion of the pro-inflammatory cytokine IL-6, leading to increased self-renewal potential of CSCs, through the activation of the JAK/STAT3 pathway [127,130]. The secretion of IL-10β and TNFα by CAAs in the tumor microenvironment has multiple effects. Firstly, the cancer cells increase their invasion and migration capacity through increased expression of matrix metalloproteinases MMPs. Secondly, the MAPK signaling pathway is activated in local endothelial cells, leading to their migration and initiation of angiogenesis. Thirdly, the secretion of IL1β also results in the local attraction of immunosuppressive neutrophils [127,130].

## 4. Adipose Cells as Delivery Platforms—Isolation, Loading and Characterization

Drug delivery cell-based platforms represent a novel therapeutic strategy that is becoming more and more popular in the medical research field. Compared to synthetic carriers that present various limitations, such as biocompatibility and biodegradability, cytotoxicity or the induction of immune response, cell-based platforms, which are live carriers, dispose of valuable targeting mechanisms and close interaction with surrounding tissues that can overcome these drawbacks. Even if both systems, synthetic or non-synthetic, pursue the same aim, to reduce the toxicity of the drugs encapsulated and to promote their therapeutic efficacy, these demands are met mostly by live cell platforms. In the case of other aspects, such as loading capacity and drug properties’ preservation, cell delivery systems experience similar challenges [19].

There are studies that are focusing on introducing adipocyte cell-based delivery platforms in cancer treatment and therapy [6,131]. At the same time, the adipocytes can be employed in other medical conditions such as regeneration and tissue engineering [132] or plastic surgery interventions [133,134].

**Figure 2:**
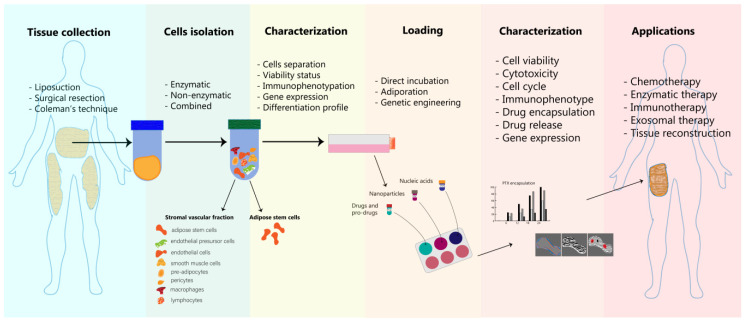
Main adipose tissue processing steps for developing adipocytes-based delivery systems with medical applicability. Once the adipose tissue is collected employing one of the three most used protocols (liposuction, surgical resection or Coleman’s technique), the next step involves cells’ isolation: enzymatic, non-enzymatic or mechanical disruption and a combination between these two. The resulted cell populations: stromal vascular fraction and adipose stem cells, undergo further characterization in order to select the proper cells according to the aim of study (most of the studies use adipose stem cells in their strategies). The loading steps refers to the encapsulation of various drugs, pro-drugs, NP formulations and also nucleic acids into adipocytes via direct incubation, adiporation or genetic engineering. The success rate of these modifications can be mainly assessed through biological and physico-chemical characterization.

Adipose stem cells are widely engaged in regenerative medicine applications due to their outstanding capacity to differentiate into multiple cell lineages (adipocytes, osteocytes, chondrocytes, cardiomyocytes and skeletal myocytes) [135]. In addition, their metabolic profile, such as angiogenic, antioxidative and immunotolerant, are described by their cytokine secretion in terms of a variety of growth factors (vascular endothelial transforming, hepatocyte, platelet-derived, placental, basic fibroblast) [136,137]. Considering the definition of a stem cell, these cells are well characterized by safety and efficiency when they are transplanted to an autologous or allogeneic host [138]. This property is one of critical importance in regenerative medicine, and not only in this domain, because all the surgery procedures or the compounds that are administered to live organisms should respect specific guidelines and ethical committee legislation. This chapter presents the main methods of adipocytes isolation, loading and characterization (Figure 2).

### 4.1. Adipocytes Isolation and Processing

In all of the cases mentioned above, the incipient state consists in isolation of adipocyte cells from the organism and maintenance in optimal conditions for further use in the healthcare line. The process of sampling the adipose tissue does not lead to surgical complications or aesthetic side effects and the amount of cells per tissue unit is usually higher than bone marrow or other tissues [135,139]. Another advantage is represented by their genetic stability [140] and resistance to senescence when cultured in vitro [141].

Usually, collected adipose tissue is composed of a variety of adipocytes, reticular fibers and blood vessels that join the adipocytes lobes together. The harvesting method is based on surgery or direct excision, but these affects the percent of viable cells. Liposuction represents a surgical technique that involves the suction of fat tissue from specific areas of the body and is well known for body contouring. Using this procedure, a high yield of adipocytes is obtained, but the issue is that this procedure affects the differentiation properties of adipose stem cells [142]. Surgical resection has the same drawback regarding the differentiation, and besides that, affects the cells’ viability. On the other hand, other surgical procedures were engaged in order to minimize this inconvenience. Coleman’s technique refers to manual suction of the fat tissue using a cannula connected to a syringe, followed by sample centrifugation and subsequent injection [143]. The strategy was developed for fat grafting purposes and is used worldwide as a gold standard for fat tissue harvesting and processing. Nowadays, this procedure can be performed by way of digital manipulation. Even so, the destruction of cells during aspiration and centrifugation steps is the main disadvantage of this technique. Ferraro et al. [144] tested different centrifugation speeds and suggested that at 1300 rpm is the optimal parameter for obtaining a good density of adipocytes, while preserving their viability and number [144]. Another cause of low yield of harvested adipocytes is represented by the donor site. Iyyaniki et al. [145] studied different sites for harvesting adipose tissue and discovered that harvesting fat from the abdominal site using direct excision and Coleman’s technique had the best results.

Adipose-derived stem cells isolation procedures are more sensitive and imply various methods. One strategy is to use an enzymatic approach by including specific enzymes and washing steps. From a clinical point of view, the most efficient enzymatic isolation consists in the use of collagenase type II for adipose tissue digestion and washing steps with ammonium chloride [135]. In 2018, Raposio and Bertozii proposed an isolation protocol for ready-to-use adipose-derived stem cells, ensuring maximum sterility in a closed circuit. Firstly, after the liposuction procedure, the adipose tissue undergoes centrifugation for discarding the serous fractions and oils. Then, an enzymatic digestion is engaged in order to release the adipose stem cells, which are double washed for purification purposes [146].

The most important part obtained after the digestion and centrifugation steps is represented by the stromal vascular fraction which is rich in adipose stem cells, endothelial precursor cells, endothelial cells, macrophages, smooth muscle cells, lymphocytes, pericytes and pre-adipocytes [147]. For this reason, this fraction undergoes further processing steps with a focus on separating cell populations. Therefore, the stromal vascular fraction is filtered in order to clean the undigested tissue and is incubated overnight at 37 °C and 5% CO_2_ in cell culture media [148]. Furthermore, it is important to analyze the viability of these cells at different time points and to monitor the cells’ behavior in culture. Regarding differences between cell types, a common strategy is to use conditioned culture media that will allow the growth of a single cell population. Rather than that, flow cytometry guarantees the distinction between cell types based on specific clusters of differentiation markers [149].

On the other hand, due to the high costs of the enzymatic methods, some groups explored mechanical isolation procedures, and they obtained promising results respecting fat grafting in skin diseases. Zhu et al. [150] compared three methods in terms of fat graft processing: gravity separation, Coleman centrifugation and washing with filtration in a closed commercially available system. Their findings suggest that the last method, washing with filtration, has gained superiority regarding cell viability and purity [150]. Another non-enzymatic innovation is represented by an intraoperative system called Lipogems, developed in Italy and patented in 2010. This system is used for harvesting, processing and injecting fat tissue and has a great applicability in regenerative medicine [151]. A different non-enzymatic approach the introduction of a new product for adipocytes isolation, named Rigenera. This system is suitable for other biological tissues’ collection and disaggregation, including dental, dermal, bone or cartilage tissue. In principle, this tool provides a minimal manipulation of the sample aiming to preserve their proper characteristics together with patient safety. The adipose tissue cells are obtained very fast via liposuction using the Berlin Autologous Lipotransfer (BEAULI) protocol, which involves water jets infiltration using different pulses [152]. Then, the lipoaspirates are subjected to the Rigenera protocol, which implies cells culture in specific growth media and mechanical desegregation [153]. De Francesco and his group compared this assay with enzymatic digestion using collagenase on the same samples. After different analysis regarding growth curves, flow cytometry, phenotype and gene expression, they concluded that this non-enzymatic protocol offers good quality results in terms of stemness and immunosuppressive properties of the isolated cells, with promising potential in micro-grafting tissue engineering [154]. Bellei et al. [155] investigated mechanical disruption using centrifugation, shaking and washing steps applied to the lipoaspiration fractions. They validated the success of these new procedures by comparison with the enzymatic digestion protocol. In this manner, they discovered an alternative for isolation of mesenchymal stem cells with good results in skin diseases therapy. On the same note, some groups combined enzymatic digestion with mechanical disruption concerning mesenchymal adipose stem cells’ isolation. Alstrup et al. [156] have obtained good results with this new strategy that reduces the amount of necessary harvested adipose tissue and the time for in vitro expansion of the cells. In this case, they decreased the amounts of collagenase without affecting the viability and the capacity to differentiate of mesenchymal stem cells. Moreover, due to the fact that the adipose tissue contains high amounts of mesenchymal stem cells, this protocol represents a better option for these cells’ isolation. Even if the enzymatical digestion method represents a gold standard in terms of isolation, it involves some risks for performing clinical trials. Unfortunately, these drawbacks are referring to variation in samples of residual enzymatic activities, which imply inconsistences and can affect the cell surface receptors involved in regeneration [157,158,159].

Among other things, a critical step after isolation is the maintenance of cells’ life and activity. In order to induce differentiation, the cells are cultured in specific growth media that can lead their differentiation toward the desired cell line. For example, the addition of dexametasone, glycerol, phosphate and ascorbic acid can lead to osteogenic differentiation [160]. For adipocytic differentiation, indomethacin, dexamethasone, hydrocortisone and insulin can be used [161]. Usually, these cells are cultured in α-modified Eagle’s medium, Dulbecco’s Modified Eagle’s medium (DMEM) and McCoy medium supplemented with 10–20% fetal bovine serum (FBS). However, for the highest safety in clinical use, it is better to avoid xenogenic compounds. A novel approach is the use of autologous plasma as a supplement instead of FBS [162].

### 4.2. Adipose Cells’ Loading Strategies

Various strategies are used for loading adipocytes with different agents, strategies that are different depending on the agents used and also their therapeutic role.

Generally, for cancer therapy, chemotherapeutic drugs are dissolved in culture media in order to be charged thorough incubation into adipocytes. Then, this cell delivery system is applied as therapy on other cells via co-culture assays [163].

Lipogems technology inspired many researchers to design novel systems for adipocytes isolation. Some of these new ideas were to include a cell culture chamber into the drain bag in order to isolate the mesenchymal stromal cells (MSCs) located here. After selection using conditioned growth media, these cells can be explored in drug delivery action by studying their ability to uptake and release chemotherapeutic drugs like paclitaxel. For this purpose, the cells are exposed to paclitaxel dissolved in culture media. After 24 h of incubation, the cells are harvested from the culture dish and are moved to another flask with fresh culture media for further use in therapeutic strategies. The efficiency of drug encapsulation depends on the effect of the drug on these cells. Paclitaxel has anti-proliferative effects and it is very important to take into consideration the inhibitory kinetics. Moreover, this aspect influences the cells’ releasing capacity. In one study, the released concentration was 10 times lower than the IC50 (half maximal inhibitory concentration) dose obtained in the case of free drug [161]. This 24 h incubation method is frequently used for paclitaxel encapsulation into adipocytes because of its advantages such as cost-effectiveness and easiness [163,164].

Different studies have focused on cell micro-vesicles for drug delivery. In this regard, Cocce et al. [165] developed an immortalized mesenchymal stromal cell line derived from adipose tissue in order to use their released micro-vesicles as carriers of paclitaxel against pancreatic cancer. The same drug was used by Wu et al. [91] who were inspired by the NP loading and delivery properties and synthesized gold nanorods functionalized with mesoporous organosilica nanospheres. The gold NPs were synthesized using the well-known seed-mediated growth method optimized by Ye et al. in 2012 [166]. The mesoporous silica nanospheres preparation followed a directed sol-gel process involving the surfactant used in gold nanorods synthesis. Then, the chemotherapeutic drug paclitaxel was dissolved in ethanol and incubated at room temperature for 24 h together with the NPs. The next day, the free paclitaxel was removed through washing steps and the NPs were loaded into mesenchymal stem cells through incubation (6 h). The un-encapsulated NPs were removed, also through washing steps. The encapsulation efficiency was facilitated by the increased surface charge of the functionalized NPs and negative charge of the cells’ membrane. The encapsulation rate increased gradually according to the time of incubation until 24 h, and after that, remained constant.

Other agents like fat acids and modified prodrugs were associated with engineer adipocytes with anticancer property in the malignant microenvironment area. In this regard, the mixture of rumenic acid and the doxorubicin reactive oxygen species responding prodrug variant were included into adipocytes growth media and the cells were exposed to it for 10 days with constant replacement every 48 h. The prodrug encapsulation efficiency was enhanced by lipid metabolism due to the addition of rumenic acid, reaching a dose of over 0.6 µg/10^6^ cells in the final day of the experiment. The releasing rate was also increased after 100 h to almost 80%. The study combines rumenic acid anticancer effects with doxorubicin in situ synthesis [6].

Therapeutic nucleic acids can also be introduced into cells through electroporation methods. In this regard, Granneman et al. [167] performed plasmid DNA electrotransfer in mature fat tissue cells with a selectivity over 99%—the technique was named “adiporation”. Fisher et al. [168] used the “adiporation” technique in order to perform DNA vaccines in a noninvasive approach by using plate electrodes. Their hypothesis was that these electrodes will efficiently concentrate the electric field within the interscapular subcutaneous adipose tissue of guinea pigs. Therefore, they performed in vivo transfection using a plasmid DNA which encodes green fluorescent protein. The group analyzed the tissue histopathology, gene expression kinetics and vaccine immunogenicity, and the results were encouraging regarding the safety of this procedure.

Adipocytes can also be genetically engineered in order to insert, modify or remove specific genes with further medical applications. One example consists in genetic manipulation of mesenchymal stem cells from adipose tissue for autoimmune disease therapy. Rostami et al. [169] used lentiviral vectors for recombinant Interleukin-23 decoy receptor transduction in an ex vivo study on these cells. The activity of the receptor involves the reduction of inflammation processes in autoimmune disorders. The transduction efficiency was over 95% and the construct was able to suppress the activity of Interleukin-7 and to induce the proliferation of Th2 cells due to Interleukin-10 enhanced expression.

### 4.3. Characterization of Engineered Adipose Tissue-Derived Cells

Physico-chemical characterization of the cell-based therapeutic systems and also biological properties such as recovery and activity during isolation and further manipulation are crucial steps in developing such platforms. Besides these investigations, another round of assays is engaged to monitor the encapsulated compounds’ efficacy. Step-by-step, the list of parameters enlarges in order to create and to achieve suitable properties in character with the study aim. As a general rule, these steps need to be followed and accomplished.

The first step of the characterization usually consists in examination of the cell populations found in the lipoaspirate complexes. The cell mixtures are supposed to follow serial processing protocols in order to select the desired population. Then, it is essential to evaluate the recovery efficiency of the cell samples. These determinations are made through various washing steps using different buffers, such as saline solutions. The viability is evaluated via trypan blue staining assay [170]. Fractionated collection versus one step collection proved significantly superior differences regarding cell number and viability rate [161].

Secondly, for the case of stem cells, it is important to evaluate their differentiation rate into specific cell lineages. These investigations are achieved through flow cytometry, immunohistochemistry and microscopy-based techniques. Flow cytometry is able to detect cell surface proteins, called clusters of differentiation (CD). Durandt et al. [171] discovered that CD36 is an important marker for the detection of adipocyte subpopulations with better efficiency than the commercial lipophilic dyes that are usually preferred for such determinations. Asc-1, PAT2 and P2RX5 are surface markers that can differentiate between brown, beige and white adipocytes due to differential expression [172]. CD10 and CD200 were proposed as differentiation markers for ASCs from different adipose depots: visceral and subcutaneous, in a screening study of 240 cell-surface markers [173]. In the case of MSCs (able to differentiate toward the adipocyte lineage), the lack of a specific marker has imposed the use of a combination of multiple positive surface molecules: CD105, CD90, CD73, CD71, CD44 and Sca-1, combined with negative markers that are expressed by endothelial and hematopoietic cells [174]. On the other hand, colorimetric assays have gained terrain due to economical and easy to use aspects. In this regard, there are various commercial dyes and optimized protocols for detecting particular differentiation hallmarks. Oil red O dye is widely used for triglycerides detection in adipocytes culture using fluorescence microscopy [175,176]. The same technique is engaged in various immunoassays for detecting specific proteins related with differentiation [175]. The novelty regarding fluorescence microscopy methods is highlighted by live-imaging platforms. De Melo et al. [177] explored fluorescence microscopy in order to assess the mineral deposition and lipid accumulation in live cultures of stem cells using Hoechst 33,258 and Bodipy 293/503 dyes. This protocol allowed for the live visualization and monitoring of the mesenchymal stem cells’ differentiation process into different cell lineages, such as osteocytes or adipocytes. Another approach is based on an automatic quantitative analysis of cell differentiation capacity. Yuan et al. [178] developed an adipocyte quantification algorithm called Fast Adipogenesis Tracking System which uses computer vision libraries for detection of adipogenesis and other types of cell lineages’ differentiation.

Third, it is important to evaluate the activity of the chemotherapeutic agents (or other agents depending on the disease) on vehicle cells. For this step, the standard methods are represented by cytotoxicity assays, such as MTT in combination with flow cytometry. The MTT test allows for the investigation of the synergistic cytotoxic effect and contributes to IC50 dose determination [179]. Flow cytometry mediated cell cycle and apoptosis analysis in order to determine the effect of drug loading on the cells. Cell cycle analysis involves propidium iodide staining of the cells’ population and shows how many of the cells are found in different cell cycle phases [180]. Apoptosis via flow cytometry also implies staining procedures using propidium iodide and Annexin V in order to detect how many of the cells are found in each cell death phase [181]. Both of these tests offer substantial information for developing such cell-based delivery systems. Furthermore, immunophenotipation at this step can reveal some significant changes concerning the cluster of differentiation panel [182]. A different assessment that can be performed at this step is called LIVE/DEAD staining (microscopy technique) [183]. It includes calcein which is able to detect live cells by staining them green and ethidium bromide which stain the dead cells with red.

Fourth, for studying the capacity to uptake and to release the therapeutic agents, the cells are cultured in the presence of the drugs for different incubation times. Then, through comparative analysis, which involves the evaluation of growth media composition at incipient and final steps, the amount of drug included in the cell is determined. Concerning the drug encapsulation and release parameters, chromatography is one of the most employed techniques, particularly high-performance liquid chromatography (HPLC) [184].

In the case of nanotransporters, such as different types of NPs that are loaded with chemotherapeutic drugs and other agents and then encapsulated into adipocytes, it is important to monitor their physico-chemical properties. In terms of genetically engineered adipocytes, the quantification of the transduction rate involves molecular biology techniques, such as PCR assays [185] and southern blot [186], and also imaging assays.

## 5. Applications of Engineered Adipose Tissue-Derived Cells in Oncology

In the last years, personalized medicine has attracted the attention of many researchers from different fields of work, especially in the area of oncology. Part of these studies is emphasizing the role of genetic engineering in the implementation of new therapeutic designs. Choi et al. [187] proposed a stem cell-based gene therapy against brainstem glioma. The group used a lentiviral vector to induce a tumor necrosis factor-related apoptosis gene into mesenchymal stem cells derived from adipose tissue. The engineered cells were then injected into the brainstems of animal mouse models and the results confirmed their tumor targeting properties and also biosafety due to the lack of side effects after 15 and 26 weeks. Other studies were inspired by interferon γ-induced protein function as a chemoattractant with increased anti-tumoral activity. Therefore, mesenchymal stem cells derived from adipose tissue can be genetically modified in order to express this protein. The study conducted by Mirzei et al. [188] described such application with favorable results in reducing tumor growth in vivo on animal models of melanoma metastases, particularly lung metastasis.

There is a consistent interest in developing enzymatic anticancer therapies using genetic engineering on mesenchymal stem cells derived from adipose tissue due to their capacity to target tumoral sites. Studies reflect that cytosine deaminase gene cell-based prodrug synthesizer construct has beneficial effects on different tumoral pathologies including: colon cancer [10], glioblastoma [189,190], prostate cancer [191,192] and gastric cancer [193]. Also, it has encouraging potential on malignant melanoma, providing long-term efficiency and over 80% survival in animal models [194]. Specifically, mesenchymal stem cells derived from adipose tissue have the property to migrate toward the tumoral sites, and based on this hypothesis, Kucerova et al. [10] developed a cell-based delivery platform by encapsulation of the CD::UPRT (cytosine deaminase) gene using retroviral transduction to create a prodrug converter. This pilot study was performed in vitro on HT-29 tumoral cells co-cultured with mesenchymal stem cells isolated from fat tissue in the presence of the prodrug 5-fluorocytosine (5-FC). The “suicide” strategy did not pose any effect on the carrier cells but had significant cytotoxicity on the cancer cells. In vivo experiment design included nude mice, subcutaneously co-injected with the treatment mixtures also containing 5-FC. In vivo expectations were achieved by tumor site targeting and inhibition of tumoral growth by systemic administration of engineered cells. In particular, molecular techniques lead to the detection of the transgene in specific organs, such as lungs and liver, in low amounts.

Li et al. [6] evaluated tumor intrinsic signaling in order to develop a mesenchymal stem cell-mediated therapy against malignant glioma. The mesenchymal stem cells derived from adipose tissue were genetically engineered using a lentiviral vector to insert a suicidal gene in response to TGF-β signaling. The experiments were performed on primary cells isolated from patients with the intention to set up a personalized design. In vitro results indicated that the aim of the study was achieved. The engineered cells have tumor targeting capacity and inhibit tumor growth by inducing apoptosis. Moreover, they present a good safety profile in vivo by prolonging the life of glioblastoma animal models when they are administered intracranially and intrathecally.

The genetic engineering methods have also directed the studies in the immunotherapy domain by building complex constructs with tumor targeting properties. Antigen-specific protein vaccines have been developed using primary adipose-derived stem cells. In cancer immunotherapy, these antigen vaccines involve tumor-specific antigens that are designed to stimulate the immune response. In vivo studies on colon and lung cancer have proven the efficiency of E7′-modified antigen inserted into adipose-derived stem cells both by co-inoculation with cancer cells and systemic administration after tumor growth [195].

Similar approaches with translational value for the oncology field are applied for other pathologies. The study of Ito et al. [196] implies a retroviral vector for human insulin cDNA transduction into primary adipocytes that were subcutaneously implanted in diabetic-induced mice. The insulin concentration is influenced by the number of injected cells and the systemic administration also assured body weight reduction. In the same context of diabetes disease, another group combined the stem cells’ advantages with gene therapy tools. Therefore, human adipocyte-derived stem cells transduction was obtained using a lentiviral vector expressing a cleavable insulin gene [128]. Tissue-specific promotors influence the gene expression pattern and can act as enhancers of such gene therapies for long-term use.

## 6. Adipocyte-Based Drug Delivery in Oncology

Adipose-derived stem cells have the ability to migrate into the tumor microenvironment and this property has a high value in drug delivery applications. Also, their isolation requires minimally invasive procedures, adding another advantage. Scioli et al. [163] adapted the autologous fat grafting for breast reconstruction for remised breast cancer patients, taking into consideration that human ASCs isolated from the patient adipose tissue can sustain the activation and proliferation of potential quiescent cancer cells. Therefore, they investigated the capacity of ASCs to load and release paclitaxel and their further effect of CG5 breast cancer cells in both in vitro (co-culture) and in vivo (xenograft) models. In both experimental setups, the paclitaxel-loaded cells were able to inhibit the proliferation and survival of breast cancer cells. Therefore, the authors concluded that these initial experiments can be translated in the clinic as a modality to maintain a local preventive environment during breast reconstruction surgery, impairing the possibility of cancer recurrence [163].

Coccè et al. [161] isolated adipose-derived MSCs with the help of a Prototype Lipogems processor (PLG-P), where the lipoaspirate is mechanically processed and the final product accumulates in a drain bag (DB). After DB-MSCs isolation and characterization, the cells were tested for the ability to uptake and release the same drug, paclitaxel, but with the intention to inhibit the activity of pancreatic carcinoma CFPAC-1 cells. The DB-MSCs primed with paclitaxel were able to mirror the effect of the free drug on cancer cell, where in the media, 10^6^ DB-ASCs/PTX liberated 140 ng of drug that corresponds to a value ten times higher than the IC50 of free paclitaxel. The uptake-release observations were in line with the parameters quantified for MSCs with other origins (e.g., bone marrow) in terms of drug delivery applications [161].

Another study compared MSCs derived from adipose tissue with the ones derived from bone marrow, both loaded with paclitaxel, on other pathologies in vitro: leukemia, osteosarcoma, neuroblastoma and prostate cancer. The results proved that MSCs derived from adipose tissue are a valuable alternative due to high efficiency in drug loading and minimal invasiveness during isolation [164].

**Figure 3:**
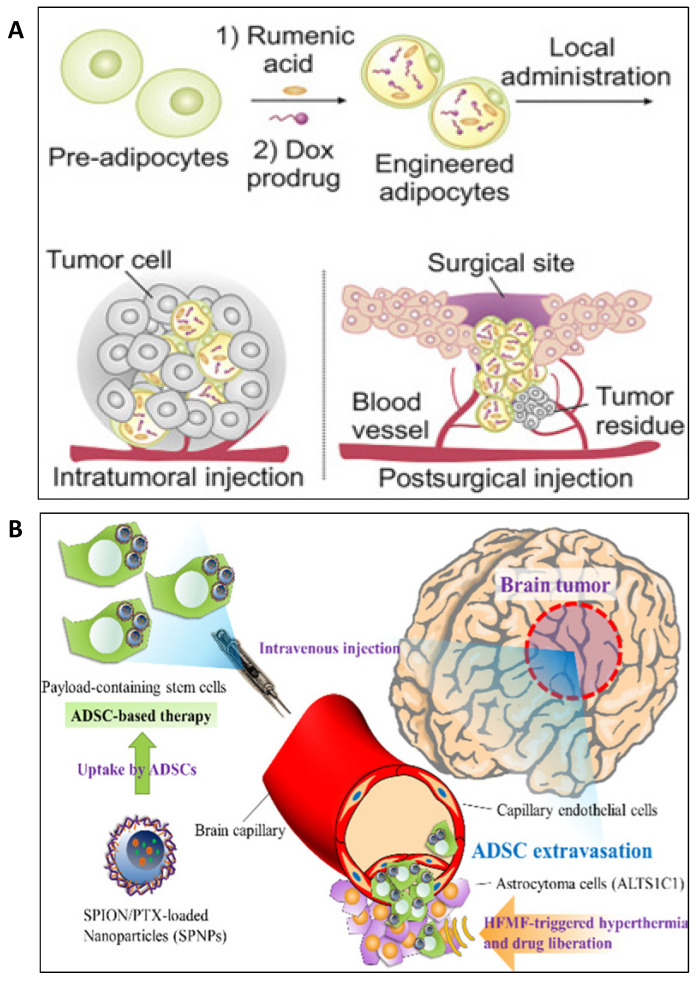
Therapeutic strategies involving adipocyte-based drug delivery. (**A**) Schematic description of pDox and RA encapsulated into adipocytes and inoculated intratumorally or within the tumor resection cavity (in vivo model: melanoma); Reprinted from *Matter*, *Cell Press*, *Vol 1*/*Issue 5*, Author(s): Di Wen, Jinqiang Wang, George Van Den Driessche, Qian Chen, Yuqi Zhang, Guojun Chen, Hongjun Li, Jennifer Soto, Ming Liu, Masao Ohashi, Zejun Wang, Peter Abdou, Quanyin Hu, Gianpietro Dotti, Song Li, Denis Fourches, Zhen Gu, Title of article: Adipocytes as Anticancer Drug Delivery Depot, Pages No.: 1203–1214, Copyright (2019), License Number 4794211061175 with permission from Elsevier [6]. (**B**) Schematic description of the ADSC-mediated delivery of SPNPs toward brain tumors for dual-modality treatment of orthotopic astrocytoma; Reprinted from *Journal of Controlled Release*, *Vol 254*, Author(s): Wen-Chia Huang, I.-Lin Lu, Wen-Hsuan Chiang, Yi-Wen Lin, Yuan-Chung Tsai, Hsin-Hung Chen, Chien-Wen Chang, Chi-Shiun Chiang, Hsin-Cheng Chiu, Title of article: Tumortropic ADSCs carrying smart nanotherapeutics for targeted delivery and dual-modality therapy of orthotopic glioblastoma, Pages No.: 119–130, Copyright (2017), License Number 4794271120645 with permission from Elsevier [12].

One of the latest studies in the field exploited the dependency of cancer cells for lipid metabolism mediated by tumor-associated adipocytes in a Trojan horse strategy (Figure 3A). Specifically, the adipocytes were engineered to encapsulate a doxorubicin prodrug (pDox) that contains a cleavable linker for reactive oxygen species (ROS) and rumenic acid (RA) that enhances the loading of the prodrug into the delivery cells. Once injected locally, the activation of lipolysis (that actually represents the Trojan horse concept) mediated the sustained release of the RA and the prodrug that was further activated through the generation of ROS. Besides the anti-tumor activity, an inhibition of PD-L1 was observed at the level of the malignant cell, subsequently enhancing the function of T cells. The in vivo validation of the therapeutic strategy was made on a B16F10 mouse melanoma model, where the survival of the animals was significantly greater, and no evident toxicity was quantified. The same treatment prevented the malignant recurrence in B16F10 mice with malignant resection [6].

## 7. Adipocyte Conjugated Nanoformulations for Drug Delivery in Oncology

Lately, the fields of nanotechnology and cell-based delivery have been combined through formulation of nanodrugs loaded into biological cells. Their application has been approached in different pathologies, including cancer.

In the context of cells derived from the adipose tissue, a complex formed of NPs payload was described that includes superparamagnetic iron oxide NP coated with oleic acid, loaded with paclitaxel (Figure 3B). The entire complex which did not exceed 110 nM in size, was encapsulated in ADSCs and experimentally adapted for treatment of malignant brain tumors. The therapeutic cargo did not exert cytotoxic effects to the host cells in the absence of stimuli. When injected into mice models of brain astrocytoma and stimulated with a high-frequency magnetic field (thermo/chemotherapy), the complex showed superior tumor inhibitory effects than the standard first line chemotherapy (temozolomide). These findings highlight the potential of adipose tissue-derived cells in overcoming the blood–brain barrier (BBB) and also selectively accumulate the therapeutic cargo at the tumor site [12]. A similar approach was used for the treatment of pancreatic cancer, where adipose-derived stem cells were loaded with pirarubicin previously encapsulated in a biodegradable polymer-nanoparticle. The biological complex was viable for approximately 48 h, time in which the pirarubicin was gradually discharged in the media with an inhibitory effect upon the proliferation rate of human pancreatic cancer cells (KP1N), together with induction of apoptosis. The results were recapitulated in mice with subcutaneous tumors through local administration [197]. Nanobiotechnology multi-strategies provide a branch of principles and techniques to transform the biosystems and to use their properties in a variety of fields. Non-viral approaches for transfection procedures have been developed using biodegradable polymers. Mangraviti et al. [198] modified the structure of poly(β-amino ester) polymer to obtain a transfection agent with higher capacity than the well-known Lipofectamine 2000. They transfected the human adipose-derived mesenchymal stem cells with bone morphogenetic protein 4 plasmid DNA obtaining a transfection efficiency of 75%. This new design therapy combines the targeting properties toward human brain tumor tissue of this protein with brain tumor targeting capacity of adipose stem cells. In vivo results concluded that intranasal administration of this construct ensures safety and success rate. A similar polyplex composed of poly(β-amino ester) polymers and pDNA but enhanced with superparamagnetic iron oxide nanoparticles (SPIONs), was tested by Balcells et al. [199] in terms of transfection efficiency, with successful results. Additionally, the incorporation of SPIONs into the transfection complex has allowed the selection of the cells containing the exogenous DNA sequence from the ones not transfected through magnetic separation.

A similar perspective of transfection design was followed in a different study. Polymeric NPs were used to engineer human adipose-derived stem cells to overexpress tumor necrosis factor-related apotosis-inducing ligand. In vivo experiments revealed that repetitive intracranial injection significantly improved the animal survival rate, achieving tumortropic migration and inhibition of the glioblastoma tumors [200].

## 8. Adipocytes-Derived Exosomes

As previously stated, the communication between the adipose tissue and other types of cells or synonymous ones is maintained partially through the secretion of exosomes charged with distinct molecular cargos. The same process is pathologically recovered in cancer [201,202,203].

Hepatocellular carcinoma (HCC) is frequently associated with chemotherapy resistance, urging toward the approach of novel therapy formulations. MicroRNA-122 (miR-122) was shown as one of the mediators of HCC chemoresistance. MiRNAs are short non-coding sequences that do not undergo protein translation, but are capable to complementarily bind different messenger RNAs (mRNAs) and inhibit their expression. These molecules have emerged as attractive therapeutic targets, especially in oncology [204,205,206,207]. In this context, mesenchymal stem cells (MSCs) derived from the adipose tissue were transfected with miR-122 plasmid and the secreted exosomes were further collected for addition upon the HCC cells. Expression analysis shows that miR-122 is effectively encapsulated in the exosomes secreted by the transfected adipose MSCs and also induces the sensitization of malignant cells to chemotherapeutic agents by modulation of miRNA target genes. In vivo data shows that injection of exosomes artificially loaded with miR-122 enhances the response to sorafenib [208].

The distribution of artificial miRNA in exosomes derived from the transfected adipose cells was investigated in detail for malignant glioma experimental therapy. MSCs were isolated from different sites: bone marrow, placenta, umbilical cord and adipose tissue, transfected with miR-124 and tested simultaneously for ability to transfer miR-124 to glioma cells in co-culture conditions through florescent labeling. The different origin MSCs showed similar capacity for delivering exogenous miRNA to the malignant cells. These similar parameters between the multi-origin MSCs in transferring the non-coding cargo toward the cancer cells, despite not being highlighted by the authors in this context, demonstrates that adipose tissue MSCs can be successfully used for such applications, but with advantages in terms of harvesting and availability. The same study shows that the mechanisms behind the miRNA transfer are attributed to both gap junctions and contact-independent delivery. Specifically, inhibition of the gap junctions by carbenoxolone determined a decrease of 50% in luciferase activity of the miR-124 plasmid target gene (SCP1-3) previously transfected into the glioma cells (compared to non-carbenoxolone cells). Contact-independent transfer was analyzed through culturing in transwell chambers that do not allow the passage of the live cells. The glioma cells transfected with the SCP-1 3′ UTR reporter gene showed a decrease of 30% in luciferase activity, a percentage smaller than in the case of the co-culture experiments; however, this demonstrated the partial role of the contact-independent transfer in delivering miRNA to target cells. This type of delivery is also mediated by exosomes, data which was revealed through co-localization of the fluorescent signals for labeled MSCs exosomes and transfected miRNA. Isolation of exosomes from miR-124-treated MSCs and control ones and incubation of glioma cells with the exosomal fraction determined an increase in the level of miRNA inside malignant cells compared to control, together with a reduction in the SCP1-3 expression level. Functional analysis showed that the MSCs-mediated transfer of miR-124 has an inhibitory role upon the migration and self-renewal capacity of glioma cells. In vivo setup reveals that the administered therapeutic MSCs are localized at the tumor peripheral zone and also inside the xenograft [209].

Exosomal content from the adipose tissue is also explored in CSCs reprogramming toward non-tumorigenic cells. Particularly, the exosomes derived from osteogenic cells differentiated from adipose-derived stem cells increase the expression of osteogenic-specific genes in cancer stem cells due to the osteogenic exosomal cargo pressure. The expression of drug resistance genes in CSCs was decreased, and the genes related to osteogenesis were enhanced, meaning that this approach can be explored to diminish therapeutic resistance [210].

## 9. Future Perspectives

At the forefront of cell-based therapies, the novel directions lead to three-dimensional (3D) cell cultures and platforms. Adipose spheroids are key players in understanding and exploring drug delivery applications in a more translational manner [211]. Importantly, the in vivo models are crucial in understating the preclinical effects of a specific experimental therapy before moving to more advanced studies in the clinic. From an anatomical point of view, the distribution of adipocytes in mouse models presents some differences when compared to humans. Rodents have two central subcutaneous pads, which are located anteriorly and posteriorly (inguinal or gluteal fat pad). The anterior pad is limited by the scapulae and axillary region. The gluteal fat pad reaches up to the dorsolumbar region, while the inguinal fat pad resembles to the gluteofemoral fat deposits in humans [212]. Lipectomy mouse models can also have many pitfalls due to the rich vascularization and innervation of mesenteric fat pads, and these factors make the surgery techniques challenging. In contrast, epididymal and retroperitoneal lipectomy shows a decrease in tumorigenesis and enhances insulin activity [213,214].

A similar importance must be also given to biomimetic strategies, which are inspired by biological features of the cells with the aim to solve disease treatment barriers [215]. Also, biomaterials have gained a lot of attention in the last years, which, coupled with cell-based delivery approaches, can be explored in medical applications, especially in tissue engineering [216]. Some of the newly developed polymers possess biodegradable features which are important in establishing safe tissue interactions and biocompatibility [217]. Synthetic biodegradable polymers are currently recommended for adipocytes’ conversion into drug delivery platforms [197].

## Figures and Tables

**Table 1:** Cells as drug delivery systems.

Cell Type	Advantages	Drug/Compound Loaded/Functionalized	Pathology	Clinical Trial Phase	Ref.
**Erythrocytes**	- Long lifespan (~120 days) [20] - Large encapsulating volume - Large surface areas - Reversible deformation [21] - Reticuloendothelial system (RES) targeting [22]	Dexamethasone Sodium Phosphate (DSP)	Ataxia Telangiectasia	Phase 2	[23]
		L-asparaginase	(a) Philadelphia Chromosome-Negative Acute Lymphoblastic Leukemia	Phase 2	[24]
			(b) Acute Myeloblastic Leukemia	Phase 2	NCT01810705
			(c) Pancreatic Cancer and Progressive Metastatic Pancreatic Carcinoma	Phase 1 and Phase 2	NCT01523808 and NCT02195180
		Glucocerebrosidase	Gaucher’s Disease		[25]
		Thymidine phosphorylase	Mitochondrial Neuro-gastrointestinal Encephalopathy (MNGIE)		[26]
		Daunorubicin	Acute Leukemia		[27]
		Doxorubicin	Lymphoma		[28]
**Platelets**	- Readily available blood cells [22] - Lifespan of 8-10 days [29] - Site-specific activation/adhesion [29]	Epidoxorubicin	Myeloma		[30]
		Doxorubicin	Lymphoma		[31,32]
		Factor VIII/Factor IX	Hemophilia		[33,34]
		ADAMTS13 (A Disintegrin and Metalloprotease with Thrombospondin Type 1 Repeats—13)	Arterial Thrombosis Associated with Thrombotic Thrombocytopenic Purpura		[35]
		Vincristine	(a) Refractory Autoimmune Hemolytic Anemia and Chronic Immune Thrombocytopenia		[36]
			(b) Immune Thrombocytopenia		[37]
**Monocytes and Macrophages**	- Can target and penetrate into inflammation sites [38] - Capable to provide the drug release in the center of tumor [39]	Protein/Peptide Antigens	Cancer		[40]
		Indocyanine Green (Contrast agent)	Inflammation		[38,41]
		Efavirenz, Ritonavir, Indinavir	Retroviral Infection		[42]
		Nano-formulated Catalase	Parkinson’s Disease		[43]
		Photosensitizer (mTHPC) and Magnetic Nanoparticles (NPs)	Cancer		[44]
**T-cells**	- Can cross biological membranes [21] - Tumor-homing - Immune response [21]	Maleimide-functionalized NPs	Prostate Cancer		[45]
		Drug (small molecules)-loaded Liposomes/Multilamellar Lipid NPs/Lipid-coated Polymer NPs	Melanoma		[46]
		Chimeric Antigen Receptor (CAR)—Anti-CD19 CAR-T	(a) Acute Lymphoblastic Leukemia (NCT03366324)	Phase 1	NCT03016377
			(b) Immune System Diseases, Immunoproliferative Disorders	Phase 2	NCT03016377
**Dendritic cells**	- They link the innate and adaptive immune responses - Can present TAAs (Tumor-Associated Antigens) to T-cells [47]	Antigen (tumor cell lysate)	Hepatocellular Carcinoma [48]		[48]
		Tumor RNA	Esophageal Squamous Cell Carcinoma [49]		[49]
		Allogeneic Apoptotic-Necrotic Melanoma Cells	Melanoma	Phase 1	NCT00515983
**Stem cells**	- Targeting capacity - Self-renewability - Can differentiate into specialized cells [20] - Homing to injured sites [21] - Can disseminate into solid tumors [50]	Pancreatic Precursor Cells	Type 1 Diabetes	Phase 2	NCT02239354
		MiR-133b	Cerebral Ischemia		[51]
		Suicide Genes	Aggressive Lung Melanoma Metastases		[52]
		Paclitaxel	Leukemia and Glioblastoma		[53,54]
		IL-12	Advanced Head and Neck Cancer	Phase 1	NCT02079324
		CCL5 Promoter	Advanced Gastrointestinal Cancer	Phase 1/2	NCT02008539

**Table 2:** General characteristics of the adipose tissue.

Important roles of adipose tissue	Expenditure of the body energy Food intake behavior Immune functions Reproduction Hematopoiesis Lymphopoiesis
Types of adipose tissue	Visceral Subcutaneous Intramuscular
Subtype of adipose tissue	White Brown Beige
Types of cells found within the adipose tissue	Adipocytes Endothelial cells Fibroblasts Connective tissue cells Pericytes Progenitor and Stem cells Macrophages Mast cells Dendritic cells Neutrophils Eosinophils Lymphocytes

**Table 3:** Highlights of the connection between adipose tissue and cancer.

Cancers linked with obesity	Endometrial cancer [80,81] Esophageal adenocarcinoma [82] Gastric cardia cancer [83] Liver cancer [84,85] Kidney cancer [86,87] Multiple myeloma [88] Meningioma [89] Pancreatic cancer [90] Gallbladder cancer [91] Breast cancer [92,93,94] Ovarian cancer [95] Thyroid cancer [96]
Molecular mechanisms behind cancer and obesity [97]	Chronic local inflammation Increased amount of estrogen High level of insulin and insulin-like growth factor-1 (IGF-1) in the blood Increased secretion of adipokines with roles in cell proliferation Indirect effect of adipocytes in modulating pathways involved in cell growth
Principal adipokines with a role in cancer progression [100]	Adiponectin Leptin
Principal signaling molecules secreted by both adipocytes and immune cells with a role in cancer progression [100]	Tumor necrosis factor alpha (TNF-α) Interleukin-6 (IL-6) Resistin Visfatin Chemokine monocyte chemoattractant protein (MCP-1) Plasminogen activator inhibitor-1 (PAI-1)
